# Gender‐related differences in spontaneous osteoclastogenesis in knee osteoarthritis: A potential peripheral biomarker for early disease progression

**DOI:** 10.1002/jeo2.70493

**Published:** 2025-10-30

**Authors:** Silvia Brogini, Giulia Sacchi, Viviana Costa, Gianluca Giavaresi, Luca Andriolo, Lorenzo Zanasi, Milena Fini, Giuseppe Filardo, Francesca Veronesi

**Affiliations:** ^1^ Surgical Sciences and Technologies IRCCS Istituto Ortopedico Rizzoli Bologna Italy; ^2^ Clinica Ortopedica e Traumatologica 2, IRCCS Istituto Ortopedico Rizzoli Bologna Italy; ^3^ Scientific Direction IRCCS Istituto Ortopedico Rizzoli Bologna Italy; ^4^ Faculty of Biomedical Sciences Università della Svizzera Italiana, Lugano Lugano‐Viganello Switzerland

**Keywords:** biomarker, gender difference, osteoarthritis, peripheral blood, spontaneous osteoclastogenesis

## Abstract

**Purpose:**

Osteoclastogenesis, the formation of bone‐resorbing osteoclasts (OCs) from peripheral blood mononuclear cells (PBMCs), is increasingly recognised as a process involved in the pathophysiology of osteoarthritis (OA). This study investigated the phenomenon of spontaneous osteoclastogenesis (OC formation without exogenous stimuli) as a potential peripheral biomarker in male and female patients with knee OA.

**Methods:**

PBMCs were isolated from 40 patients with knee OA (20 males, 20 females; Kellgren–Lawrence [KL] Grades I–II) and cultured for 21 days without osteoclastogenic factors. OC viability (Alamar Blue assay), percentage of mature OCs (tartrate‐resistant acid phosphatase [TRAP] histochemical staining) and secretion of Cathepsin K (CTSK) and matrix Metalloproteinases (MMPs) 7 and 9 (enzyme‐linked immunosorbent assay [ELISA] tests) were assessed.

**Results:**

Demographic and clinical characteristics are comparable between male and female patients. OC viability was also similar between groups (*p* = 0.494). However, males exhibited a significantly higher percentage of mature OCs compared to females (*p* < 0.001). ELISA analysis revealed significantly higher levels of MMP7 and MMP9 in males (*p* < 0.001 for both), while CTSK levels did not differ significantly between groups (*p* = 0.136).

**Conclusions:**

This study provides the first evidence of spontaneous osteoclastogenesis in OA patients, even at early stages, and highlights significant sex‐related differences in OC maturation and protein secretion. These findings suggest that spontaneous osteoclastogenesis may reflect systemic dysregulation of bone resorption and could serve as a potential biomarker for OA progression. Larger, controlled studies are needed to validate its diagnostic and prognostic value.

**Level of Evidence:**

Level I.

**Abbreviations**
BMI: body mass index
CI: confidence interval
CTSK: Cathepsin K
ELISA: enzyme‐linked immunosorbent assays
FCS: fetal calf serum
IQR: interquartile range
KL: Kellgren–Lawrence
MMP7: matrix Metalloproteinase 7
MMP9: matrix Metalloproteinase 9
M‐CSF: macrophage‐colony stimulating factor
OA: osteoarthritis
OCs: osteoclasts
PBMCs: peripheral blood mononuclear cells
PBS: phosphate‐buffered saline
PTH: parathiroid hormone
RANKL: receptor activator of nuclear factor kappa‐Β ligand
ROIs: regions of interest
TNF‐α: tumour necrosis factor‐alpha
TRAP: tartrate‐resistant acid phosphatase

## INTRODUCTION

Osteoarthritis (OA) is a multifactorial joint disease affecting millions of individuals worldwide [24, 34]. Current diagnostic approaches rely primarily on clinical evaluation and imaging techniques, which are limited in their ability to detect early‐stage OA and to effectively monitor disease progression [22, 27, 38]. In this context, the identification of reliable and minimally invasive biomarkers has emerged as a crucial goal in OA research [1, 4, 11]. Several candidate biomarkers have been proposed and are commonly classified according to tissue metabolism (bone, cartilage and synovial markers), pathological pathways (inflammatory and genetic markers) and biological function (chemokines, growth factors, acute‐phase proteins, etc.) [5]. However, their clinical applicability remains limited, and no widely accepted biomarker has yet been established for routine use.

Given this context, the present study focused on osteoclasts (OCs) as candidate biomarkers, as their activity is increasingly implicated in subchondral bone remodelling [8, 13, 14, 16, 21, 23, 25, 32, 35]. Investigating this process may offer a novel, non‐invasive approach to detect and monitor OA.

To date, only one in vitro study has reported the formation of mature OCs derived from peripheral blood mononuclear cells (PBMCs) of patients with OA, cultured for 21 days in the presence of osteoclastogenic stimuli such as receptor activator of nuclear factor κB ligand (RANKL) and macrophage colony‐stimulating factor (M‐CSF) [9]. This finding may be explained by the dysregulation of bone remodelling that occurs particularly in the early stages of OA, characterised by enhanced osteoclastic activity, which contributes to subchondral bone loss, osteophyte formation and cartilage degradation [13, 14, 21, 25, 32].

Otherwise, spontaneous osteoclastogenesis refers to the in vitro formation of OCs in the absence of exogenous osteoclastogenic stimuli. This phenomenon has previously been observed in some musculoskeletal conditions such as cancer‐related bone disease, osteoporosis and inflammatory skeletal disorders [29, 30, 31, 34], but it has never been studied in the context of OA. The presence of spontaneous OC formation may reflect systemic alterations in inflammatory and bone metabolic pathways, potentially driven by elevated circulating mediators such as RANKL, tumour necrosis factor‐alpha (TNF‐α) or parathyroid hormone (PTH) [29, 30].

Given these premises, the present study investigates whether spontaneous osteoclastogenesis occurs in patients with knee OA, aiming to explore its potential role as a peripheral biomarker of disease activity. To better understand its biological relevance, gender‐related differences in this phenomenon were evaluated. The hypothesis was that spontaneous osteoclastogenesis could be observed in OA patients, indicating an intrinsic or systemic dysregulation of bone resorption mechanisms and offering a potential gender‐specific biomarker for joint degeneration.

## MATERIALS AND METHODS

### Study design

Patients were enrolled from an ongoing randomised controlled trial (RCT) on the injective treatment of knee OA, whose inclusion and exclusion criteria are reported in Table 1 [2]. The study was approved, on 23 May 2023, by the Ethical Committee (CE AVEC: 150/2023/Sper/IOR). Informed consent was obtained from each patient for study participation.

**Table 1:** Inclusion and exclusion criteria of included patients.

Inclusion criteria	Exclusion criteria
History of chronic pain or swelling (at least 6 months); Age 40−75 years; KL Grade I–II; No benefit after at least 4 months of nonoperative treatment	Age < 40 and >75 years; Major coronal deviation >5°; History of trauma or intra‐articular injection therapy within 6 months before treatment; Knee surgery within 12 months; Presence of any concomitant knee lesion causing pain or swelling other than OA; Neoplasm, infections, systemic disorders

Abbreviations: KL, Kellgren–Lawrence; OA, osteoarthritis.

### Demographic and clinical patient characteristics

The 40 patients were enrolled over a 4‐month period, and their peripheral blood samples (~2 mL) were obtained from each patient before treatment and sent, in sterile conditions, to the laboratory.

### Spontaneous osteoclastogenesis evaluations

**Figure 1:**
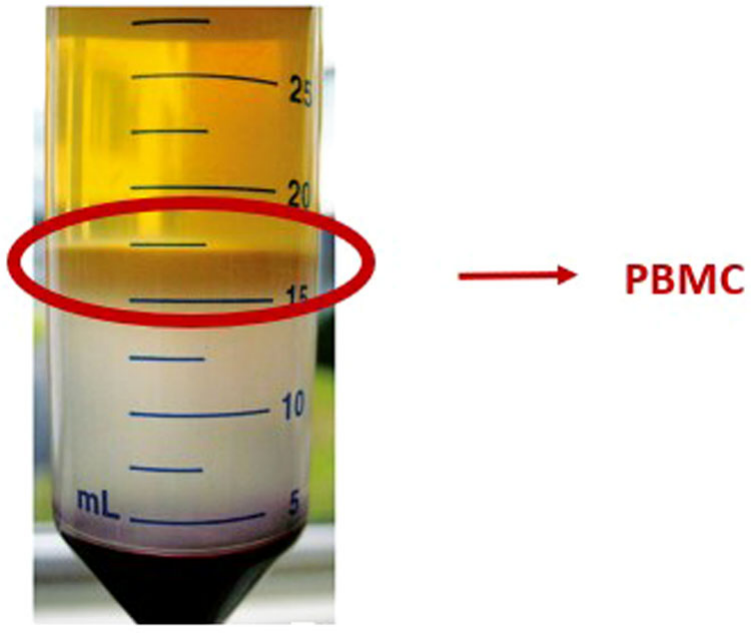
Representative image of peripheral blood mononuclear cell (PBMC)'s isolation by density gradient with Ficoll.

PBMCs were obtained from peripheral blood samples according to the Ficoll (Sigma‐Aldrich, St. Louis, MO, USA) method (Figure 1). Briefly, peripheral blood was diluted with phosphate‐buffered saline (PBS, Sigma‐Aldrich, MO, USA) (ratio 1:1) and the resulting quantity was layered on Histopaque 1077 (ratio 2:1). PBMCs were obtained through density gradient centrifugation for 30 min at 700 × *g*, after that the interface between plasma and Ficoll, that contained PBMCs was collected, washed and resuspended in Dulbecco's modified Eagle's medium (DMEM; Sigma‐Aldrich, MI, USA) without fetal calf serum (FCS). Cells were then counted and seeded at a density of 1.5 × 10^6^/cm^2^ in a 24‐well plate, and the medium was refreshed every 3 days. After 3 days of culture, the non‐adherent cells were removed, while the adherent cells (PBMCs) were evaluated through a light microscope (Olympus IX 71) and the images were acquired with a digital image capture system (×20 objective and an Olympus XC camera) (Boston Industries, Inc., 10 Industrial Rd., Walpole, MA 02081, USA).

*OC viability*. After 21 days of culture, cell viability was evaluated using the Alamar Blue dye assay (Serotec, Oxford, UK). Briefly, the dye was added to the culture wells at a 1:10 volume ratio and incubated for 4 h at 37°C. Then, the absorbance of the supernatants was measured spectrophotometrically at 570‐ and 600‐nm wavelengths using a MicroPlate reader (BioRad, Hercules, CA, USA).

*OC percentage*. After 21 days of culture, tartrate‐resistant acid phosphatase (TRAP) histochemical staining was performed to identify and count mature OCs, following the manufacturer's instructions (Sigma, St. Louis, MO, USA). Cells were considered mature OCs if they appeared large, multinucleated (≥3 nuclei), and stained dark purple [20]. Images were acquired with a standard light microscope (Olympus IX71) equipped with a digital camera (XCell, Olympus Italia Srl, Segrate Milano, Milano, Italy) at ×20 magnification. The percentage of OCs was calculated by dividing the number of OCs by the total number of cells, within 5 regions of interest (ROIs) at ×20 of magnification and then multiplying the result by 100.

*Protein production*. After 21 days of culture, the supernatants from OC cultures were collected, centrifuged to remove particulates and stored at −20°C. Immunoenzymatic assays (enzyme‐linked immunosorbent assay, ELISA) were employed to quantify the levels of key proteins secreted by OCs: Cathepsin K (CTSK, ng/mL), Matrix Metalloproteinase 7 (MMP7, ng/mL) and MMP9 (ng/mL) (Cloud‐Clone Corp., TX 77494, USA), according to the manufacturer's instructions. Absorbance was measured at 450 using an ELISA reader (Imark Microplate Reader, ELISA‐Biorad SRL). Each sample was analysed in triplicate.

### Power analysis and statistical analysis

Sample size estimation was performed using G*Power v. 3.1.9.4. Assuming a large effect size (Cohen's *d* = 0.86) for differences in spontaneous osteoclastogenesis between males and females, with a significance level of 0.05 and statistical power of 0.80, the required sample size was 20 individuals per group, calculated using a two‐tailed *t*‐test for independent samples.

All statistical analyses were performed using GraphPad Prism (version 9.0.0, GraphPad Software, San Diego, CA, USA). Due to the non‐parametric distribution of some data, comparisons between the two experimental groups (male vs. female donors) were performed using the Mann–Whitney *U* test. Data are reported as median values with interquartile ranges (IQRs), and box‐and‐whisker plots were used to graphically represent the data distribution. Sample sizes for each group are indicated in the respective figure legends. A *p*‐value of less than 0.05 was considered statistically significant.

## RESULTS

Demographic features of the 40 patients (20 females and 20 males) were summarised in Table 2. No statistically significant differences were found between males and females in terms of age (*p* = 0.123) and KL grade distribution (*p* = 0.530), whereas body mass index (BMI) differed significantly (*p* = 0.011).

**Table 2:** Included patient characteristics (mean ± standard deviation [SD] [95% confidence interval (CI)]).

Characteristic	Female (*n* = 20)	Male (*n* = 20)	*p* value
Age (years)	56.0 ± 3.5 [52.4–59.5]	58.8 ± 3.1 [55.7–61.8]	0.123
BMI (kg/m²)	24.3 ± 3.7 [22.4–26.1]	26.5 ± 2.2 [25.5–27.6]	0.011
KL grade	KL I = 9; KL II = 11	KL I = 11; KL II = 9	0.530

Abbreviations: BMI, body mass index; KL, Kellgren–Lawrence.

**Figure 2:**
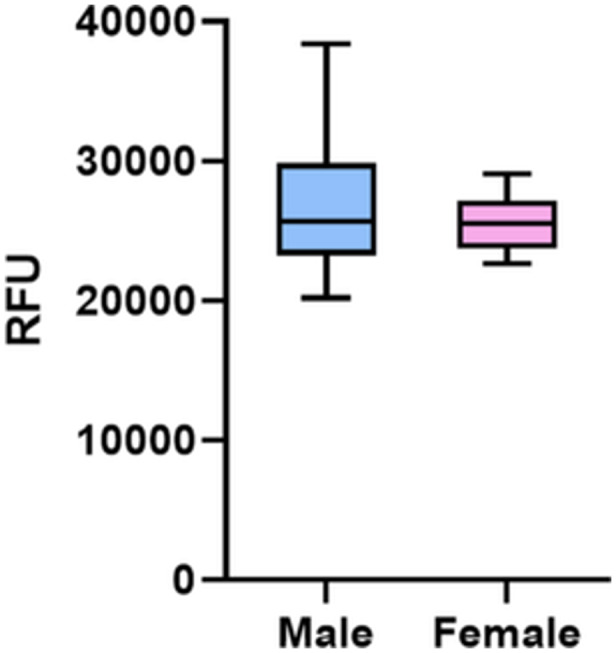
Box plot (median, interquartile range [IQR]) shows osteoclast (OC) viability (Alamar Blue assay) in male and female patients. Mann–Whitney test (not significant [NS]). RFU, relative fluorescence units.

The results of OC viability are presented in Figure 2. The analysis revealed no significant differences between males and females in viability (*p* = 0.494). The mean viability was 26876 ± 4608 relative fluorescence units (RFU) in males (range: 20188–38370 RFU) and 25521 ± 2032 RFU in females (range: 22642–29070 RFU) (Table 3).

**Table 3:** Results of osteoclast (OC) viability, tartrate‐resistant acid phosphatase (TRAP)‐positive OCs, Cathepsin K (CTSK), matrix Metalloproteinase 7 (MMP7) and MMP9 in males and females.

Outcomes	Groups	Mean ± SD	Range (min–max)	*p* value
OC viability (RFU)	Males (*n* = 20)	26876 ± 4608	20,188–38,370	*p* = 0.494
	Females (*n* = 20)	25,521 ± 2032	22,642–29,070	
% TRAP‐positive OCs	Males (*n* = 20)	42 ± 7	30–55	*p* < 0.001
	Females (*n* = 20)	30 ± 8	20−50	
CTSK (ng/mL)	Males (*n* = 20)	429 ± 43	346–495	*p* = 0.136
	Females (*n* = 20)	447 ± 26	410–490	
MMP7 (ng/mL)	Males (*n* = 20)	354 ± 31	200–298	*p* < 0.001
	Females (*n* = 20)	199 ± 45	106–299	
MMP9 (ng/mL)	Males (*n* = 20)	42 ± 5	30–49	*p* < 0.001
	Females (*n* = 20)	36 ± 5	26–44	

Abbreviations: max, maximum; min, minimum; RFU, relative fluorescence units; SD, standard deviation.

**Figure 3:**
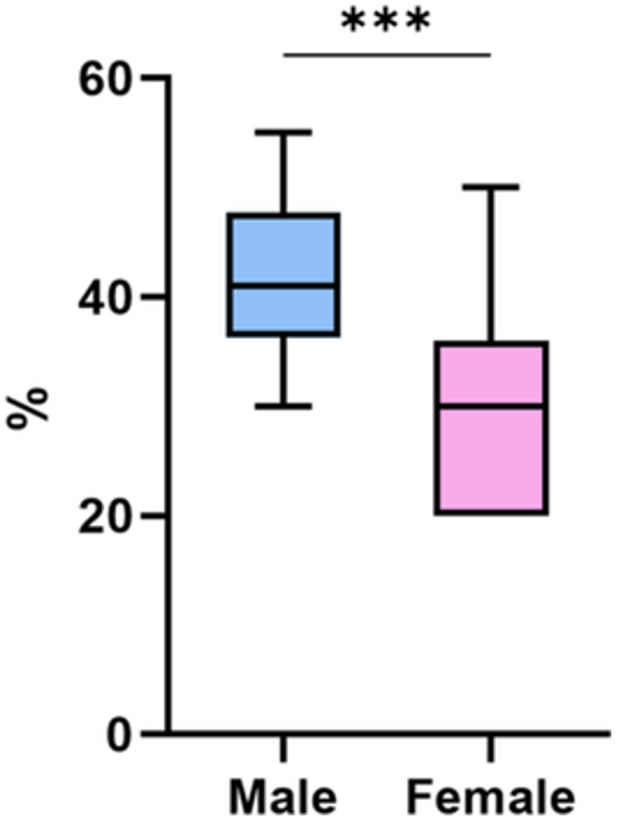
Box plot (median, interquartile range [IQR]) showing the percentage of osteoclasts (OCs) in male and female samples. Mann–Whitney test (***p < 0.001). % = OC percentage.

**Figure 4:**
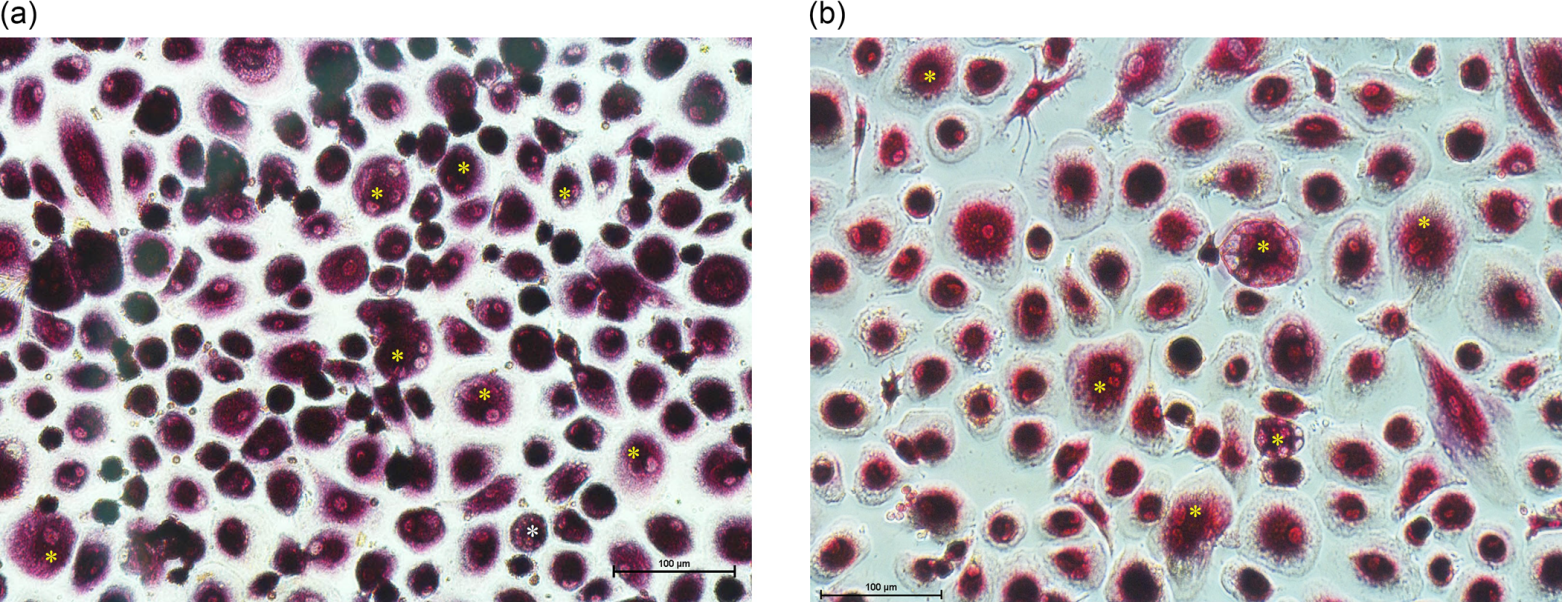
Representative images of tartrate‐resistant acid phosphatase (TRAP) staining. Magnification 20×. Scale bar: 100 µm. Asterisk: osteoclast. (a) Osteoclasts (OCs) from male patients and (b) OCs from female patients after 3 weeks of culture without osteoclastogenic stimuli.

As shown in Figures 3 and 4, the results showed that men exhibited a significantly higher percentage of OCs compared to women (42% ± 7%, range: 30%–55% for males; 30% ± 8%, range: 20%–50% for females) (*p* < 0.001) (Table 3). From a morphological perspective, mature OCs had more nuclei, mostly located centrally, a larger cell size compared to undifferentiated cells, an active ruffled border and a clear zone surrounding the cell membrane. No significant differences were observed in OC size or nuclear number between males and females (median nuclei per OC: males 4 [IQR 3–5] vs. females 4 [IQR 3–5], *p* = 0.842; cell size: males 2450 µm² [IQR 2200–2700] vs. females 2380 µm² [IQR 2150–2600], *p* = 0.733).

**Figure 5:**
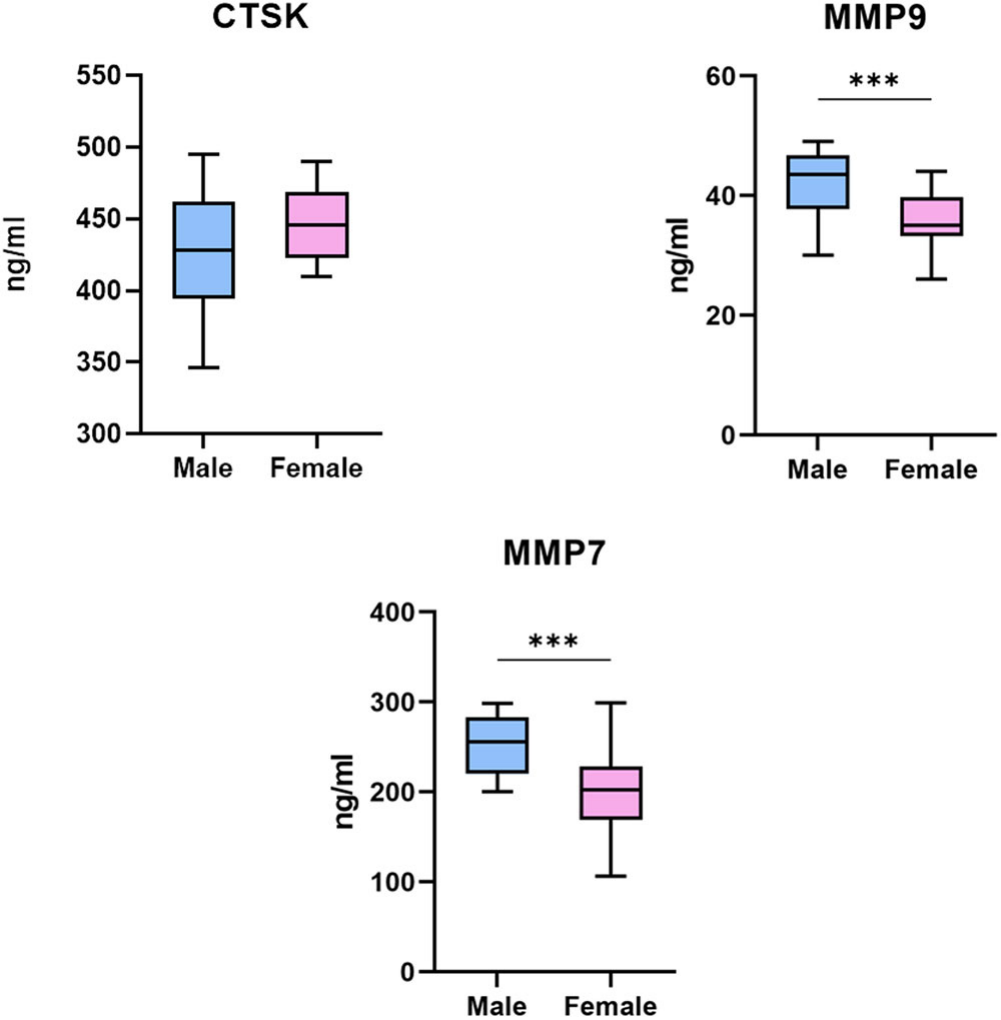
Box plot (median, interquartile range [IQR]; *n* = 20) of Chatepsin K (CTSK), matrix Metalloproteinase 7 (MMP7) and matrix Metalloproteinase 9 (MMP9) production. Mann–Whitney test (***p < 0.001).

Figure 5 shows the results of the ELISA assays performed to assess the production of different proteins produced by mature OCs. Significant differences were detected in the expression of MMP7 and MMP9 between males and females (*p* < 0.001). No significant differences were observed for CTSK protein (*p* = 0.136).

## DISCUSSION

This study provides the first evidence that spontaneous osteoclastogenesis occurs in patients with knee OA, even in the early stages of the disease, and reveals significant gender‐related differences in OC activity and protein secretion, with men exhibiting a significantly higher percentage of OCs and greater MMP7 and MMP9 production compared to women. The age range of 40–75 years was deliberately chosen to ensure both clinical and biological relevance. Epidemiological data indicate that this population is the most affected by knee OA, making the findings particularly applicable to the typical patient demographic. OA onset before the age of 40 is relatively uncommon and is often linked to secondary causes such as trauma or congenital abnormalities [15]. In contrast, individuals over 75 frequently present with complex comorbidities that may influence systemic inflammatory responses and cellular behaviour, particularly in in vitro models involving PBMCs. This selection helped minimise confounding factors and enhanced the interpretability of spontaneous osteoclastogenesis within the OA‐specific context [28].

In literature, spontaneous osteoclastogenesis was observed in other musculoskeletal pathologies [29, 30, 31, 34], but not in OA.

Previous literature has demonstrated that OC‐mediated bone resorption contributes to subchondral bone alterations and cartilage degeneration in OA [8, 13, 14, 16, 21, 23, 25, 32, 35], and, for the first time, in 2013, Durand et al. observed higher in vitro OC viability and activity when OA PBMCs were cultured in the presence of osteoclastogenic stimuli, in comparison to healthy PBMCs [9].

This study is the first report documenting spontaneous differentiation under basal conditions, suggesting a preactivated or primed state of circulating precursors in OA patients, potentially reflecting an inflammatory systemic milieu or disease‐related epigenetic programming [29, 30]. The findings extend the current understanding by suggesting that systemic pro‐osteoclastogenic signals may be intrinsically present in OA, potentially contributing to joint degeneration.

Considering that men and women presented the same level of early OA in this study, this suggests that men may have an inherently greater osteoclastogenic activity, which could contribute to more aggressive bone resorption and OA progression.

The analysis of protein production also revealed that MMP7 and MMP9 levels were significantly higher in male patients. OCs express and secrete several proteases, including MMP7 and MMP9, which are the primary osteoclastic enzymes involved in extracellular matrix degradation and in preparing the resorption microenvironment [17, 24]. MMP7 processes osteopontin and may facilitate OC activation even in the absence of strong direct osteoclastogenic signals, while MMP9 is critical for the removal of unmineralised bone matrix [18, 37]. The increased expression of MMPs in men may indicate a greater degree of matrix remodelling and tissue breakdown, which reflects the increased resorptive activity in male‐derived OCs found in the literature [6].

CTSK levels did not show significant gender‐related differences, suggesting that its regulation may not be gender‐dependent in OA patients. The results obtained are consistent with the literature [36, 37]. In particular, the absence of differences in CTSK expression, alongside a marked increase in MMP7 and MMP9 levels, can be interpreted considering compensatory mechanisms described in CTSK‐deficient animal models, in which OC activity is maintained through the induction of alternative MMPs [37]. Furthermore, CTSK appears to act as an epigenetic regulator, modulating the activation of osteoclastic genes. Zhu et al. demonstrated that the deletion of both CTSK and MMP9 leads to severe osteopetrosis due to impaired OC activation and function resulting from the loss of their compensatory roles. CTSK activity may not be necessary or may even be suppressed in less acidic inflammatory environments, such as peripheral blood, where MMP9 could be independently activated [36]. Interestingly, MMP9 can also be activated by CTSK in acidic environments, but this pathway may not be dominant in peripheral blood, thereby explaining the observed dissociation [7]. Moreover, in vitro studies have shown that inflammatory activity can directly modulate MMP9 expression in peripheral blood‐derived OCs, confirming the potential role of the systemic inflammatory microenvironment in determining the activity of these enzymes [12]. These findings underscore the importance of considering sex as a key biological variable in OA research. The observed differences may have implications for personalised treatment approaches, as males and females may respond differently to therapeutic interventions targeting OC activity. The gender‐related differences observed could be explained as follows. First, the study showed that men have a higher percentage of OCs and higher levels of MMP7 and MMP9 enzymes involved in extracellular matrix degradation. This could suggest that the bone resorption process is more active in men, potentially contributing to a faster disease progression. Women, despite having a higher incidence of OA, may have a less aggressive response in terms of bone destruction. Similarly, Li et al. analysed femoral heads from 110 OA patients and reported that bone remodelling in subchondral trabecular bone increased with age in men but not in women [19]. In addition, oestrogens have a protective effect on bone and cartilage tissues, inhibiting excessive OC formation and reducing inflammation [3]. Although postmenopausal women experience greater bone loss, individuals within the 40–75 years age range may still retain partial oestrogenic protection in subchondral bone compared to men. In contrast, men lack this hormonal advantage, which may make them more susceptible to bone degradation. Finally, the increased expression of MMP7 and MMP9 in men may reflect a more pronounced inflammatory state, accelerating osteoclastogenesis. Pan et al. highlighted that gender‐specific responses in OA involve differential production of MMPs and inflammatory cytokines in joint tissues, with male‐derived cells more responsive to catabolic stimuli [26]. Females might possess different compensatory mechanisms that mitigate the OA progression. Accordingly, men may experience greater bone destruction due to enhanced spontaneous OC activation, whereas in women, the bone resorption mechanisms may be less aggressive.

The identification of spontaneous osteoclastogenesis in OA patients has important clinical implications. First, the detection of this phenomenon, even in early‐stage OA, underscores its potential utility as a non‐invasive peripheral biomarker for the early diagnosis and monitoring of disease progression. The ability to detect active osteoclastogenesis from PBMCs without the need for exogenous stimuli may provide a valuable tool for identifying patients at higher risk of rapid structural joint deterioration. Moreover, the observed gender‐related differences in OC activity and MMP expression highlight the importance of considering gender as a biological variable in both diagnostic and therapeutic strategies. These differences suggest that personalised treatment approaches targeting OC‐mediated bone resorption may be more effective when tailored to the patient's gender [33]. For example, male OA patients may benefit more from early intervention with OC‐targeting agents, given their higher baseline osteoclastogenic activity and proteolytic enzyme production.

From a therapeutic perspective, these findings support the exploration of anti‐resorptive therapies, such as RANKL inhibitors (e.g., denosumab) or bisphosphonates, in selected OA patient populations. While these agents are primarily used for osteoporosis, their application in OA could potentially limit subchondral bone degradation, reduce osteophyte formation and delay cartilage damage, particularly in the early stages of the disease when bone resorption predominates.

Ultimately, these findings may pave the way for a biomarker‐guided, patient‐tailored approach to OA management, integrating diagnostic, prognostic, and therapeutic strategies centred on OC biology.

Despite the novel insights offered by this study, some limitations must be acknowledged. The sample size was relatively small (*n* = 40), which may limit the generalisability of the findings to the broader OA population. These evaluations should be performed in larger studies to confirm these findings. In addition, a further limitation of this study is the absence of a healthy control group. However, preliminary assessments and existing literature [29, 30, 31] indicate that PBMCs from healthy individuals do not survive beyond three days in culture without osteoclastogenic stimuli, thereby preventing any measurable osteoclastogenesis or protein secretion after 21 days. This makes direct comparison technically unfeasible. In addition, the inclusion of both premenopausal and postmenopausal women within the age range could represent a confounding factor. Finally, although BMI differed significantly between male and female patients (*p* = 0.011), it is unlikely to have substantially influenced our main findings for the following reasons: (1) Both groups were within the non‐obese BMI range (females: 24.3 ± 3.7 kg/m²; males: 26.5 ± 2.2 kg/m²), minimising the potential impact of BMI‐related metabolic alterations on PBMCs behaviour; (2) current evidence suggests that osteoclastogenesis is more strongly driven by inflammatory and hormonal factors rather than by mild differences in BMI within this range [10].

Despite this limitation, the study highlights the presence of spontaneous osteoclastogenesis in OA patients, with gender‐specific patterns that could represent both a novel biomarker and a potential therapeutic target.

## CONCLUSIONS

This study documented the presence of spontaneous osteoclastogenesis in OA patients, highlighting significant gender‐related differences in terms of OC percentage and protein production. These results contribute to the growing body of evidence supporting a possible involvement of OC in the etiopathogenetic disease processes and its potential role as a peripheral biomarker in early OA diagnosis, progression monitoring and possibly a target for future treatments to address OA.

## AUTHOR CONTRIBUTIONS

*Conceptualisation*: Giuseppe Filardo and Milena Fini. *Methodology*: Giulia Sacchi and Lorenzo Zanasi. *Formal analysis and investigation*: Silvia Brogini and Gianluca Giavaresi. *Writing—original draft preparation*: Francesca Veronesi and Luca Andriolo. *Writing—review and editing*: Francesca Veronesi and Silvia Brogini. *Funding acquisition*: Luca Andriolo. *Supervision*: Francesca Veronesi.

## CONFLICT OF INTEREST STATEMENT

The authors declare no conflicts of interest.

## ETHICS STATEMENT

All procedures performed in studies involving human participants were in accordance with the ethical standards of the institutional and/or national research committee and with the 1964 Helsinki Declaration and its later amendments or comparable ethical standards. The study was approved on 23 May 2023 by the local Ethical Committee (CE AVEC: 150/2023/Sper/IOR).

## PATIENT CONSENT STATEMENT

Informed consent was obtained from all individual participants included in the study.

## PERMISSION TO REPRODUCE MATERIAL FROM OTHER SOURCES

NA.

## FOR CLINICAL TRIALS

The trial was registered at ClinicalTrials.gov (registration No. NCT06040957).

## Data Availability

The data that support the findings of this study are available from the corresponding author upon reasonable request.
